# Supplementary material for: Mechanisms governing the pioneering and redistribution capabilities of the non-classical pioneer PU.1
Source: Nat Commun. 2020 Jan 21;11:402. doi: 10.1038/s41467-019-13960-2 (PMC6972792; doi:10.1038/s41467-019-13960-2)
Supplement: Supplementary file 7 — Source data [file 41467_2019_13960_MOESM7_ESM.zip › Source_Data/FigureS5/FigureS5A_MotifsCommonPeaks/homerResults/motif10.similar.html]

motif10

## Information for motif10

T
A
C
G
T
C
A
G
A
T
G
C
T
G
A
C
C
T
A
G
A
T
C
G
G
T
C
A
C
A
G
T
T
A
C
G
A
G
T
C
A
C
T
G
T
C
A
G
  
Reverse Opposite:  

A
G
T
C
T
G
A
C
T
C
A
G
A
T
G
C
G
T
C
A
C
A
G
T
T
A
G
C
G
A
T
C
A
C
T
G
T
A
C
G
A
G
T
C
A
T
G
C
  

|  |  |
| --- | --- |
| p-value: | 1e-164 |
| log p-value: | -3.793e+02 |
| Information Content per bp: | 1.537 |
| Number of Target Sequences with motif | 845.0 |
| Percentage of Target Sequences with motif | 9.51% |
| Number of Background Sequences with motif | 966.1 |
| Percentage of Background Sequences with motif | 2.54% |
| Average Position of motif in Targets | 101.6 +/- 48.3bp |
| Average Position of motif in Background | 100.3 +/- 63.7bp |
| Strand Bias (log2 ratio + to - strand density) | -0.1 |
| Multiplicity (# of sites on avg that occur together) | 1.04 |
| Motif File: | file (matrix) reverse opposite |

### Similar de novo motifs found

|  |  |  |  |  |  |  |  |
| --- | --- | --- | --- | --- | --- | --- | --- |
| Rank | Match Score | Redundant Motif | P-value | log P-value | % of Targets | % of Background | Motif file |
| 1 | 0.958 | T A C G C A T G T C G A A T G C G T A C C T A G A C T G G T C A A C G T A C T G G A T C | 1e-135 | -311.920379 | 5.89% | 1.15% | motif file (matrix) |
| 2 | 0.838 | A T C G T A G C A G T C G T A C C G T A T A G C G T C A A G C T G T A C G A T C A C T G A T C G A G T C | 1e-108 | -249.205302 | 3.51% | 0.44% | motif file (matrix) |
| 3 | 0.843 | C G T A A G T C C G T A A C G T A G T C A G T C A C T G A C T G T C A G C T A G | 1e-25 | -57.917206 | 0.71% | 0.07% | motif file (matrix) |
